# How safe is prehospital care? A systematic review

**DOI:** 10.1093/intqhc/mzab138

**Published:** 2021-10-08

**Authors:** Paul O’connor, Roisin O’malley, Kathryn Lambe, Dara Byrne, SinÉad Lydon

**Affiliations:** Discipline of General Practice, School of Medicine, National University of Ireland Galway, 1 Distillery Road, Galway H91 TK33, Ireland; Irish Centre for Applied Patient Safety and Simulation, National University of Ireland Galway, Co. Galway H91 TK33, Ireland; Discipline of General Practice, School of Medicine, National University of Ireland Galway, 1 Distillery Road, Galway H91 TK33, Ireland; Irish Centre for Applied Patient Safety and Simulation, National University of Ireland Galway, Co. Galway H91 TK33, Ireland; Health Research Board, 67-72 Lower Mount Street, Dublin D02 H638, Ireland; Irish Centre for Applied Patient Safety and Simulation, National University of Ireland Galway, Co. Galway H91 TK33, Ireland; School of Medicine, National University of Ireland Galway, Co. Galway H91 TK33, Ireland; Irish Centre for Applied Patient Safety and Simulation, National University of Ireland Galway, Co. Galway H91 TK33, Ireland; School of Medicine, National University of Ireland Galway, Co. Galway H91 TK33, Ireland

**Keywords:** prehospital, systematic review, patient safety incident, harm

## Abstract

**Background:**

As compared to other domains of healthcare, little is known about patient safety incidents (PSIs) in prehospital care. The aims of our systematic review were to identify how the prevalence and level of harm associated with PSIs in prehospital care are assessed; the frequency of PSIs in prehospital care; and the harm associated with PSIs in prehospital care.

**Method:**

Searches were conducted of Medline, Web of Science, PsycInfo, CINAHL, Academic Search Complete and the grey literature. Reference lists of included studies and existing related reviews were also screened. English-language, peer-reviewed studies reporting data on number/frequency of PSIs and/or harm associated with PSIs were included. Two researchers independently extracted data from the studies and carried out a critical appraisal using the Quality Assessment Tool for Studies with Diverse Designs (QATSDD).

**Results:**

Of the 22 included papers, 16 (73%) used data from record reviews, and 6 (27%) from incident reports. The frequency of PSIs in prehospital care was found to be a median of 5.9 per 100 records/transports/patients. A higher prevalence of PSIs was identified within studies that used record review data (9.9 per 100 records/transports/patients) as compared to incident reports (0.3 per records/transports/patients). Across the studies that reported harm, a median of 15.6% of PSIs were found to result in harm. Studies that utilized record review data reported that a median of 6.5% of the PSIs resulted in harm. For data from incident reporting systems, a median of 54.6% of incidents were associated with harm. The mean QATSDD score was 25.6 (SD = 4.1, range = 16–34).

**Conclusions:**

This systematic review gives direction as to how to advance methods for identifying PSIs in prehospital care and assessing the extent to which patients are harmed.

## Introduction

In recent years, prehospital care has become an integrated part of the healthcare system where advanced care is provided to critically ill and injured patients [1]. Prehospital care can be defined as the care received by a patient from an emergency medical service before arriving at a hospital [2]. Prehospital care is potentially hazardous with the possibility for patients to experience a patient safety incident (PSI; defined as any unintended or unexpected incident(s) that could have or were judged to have led to patient harm [3, 4]). However, as compared to primary care (healthcare provided by general practitioners or other healthcare professionals to whom a patient has direct access [2]) and secondary care (care usually provided in a hospital setting [2]), little is known about PSIs in prehospital care settings.

In secondary care it is estimated that between 4% and 17% of hospital admissions are associated with a PSI [5], with 7% resulting in death [6]. Specifically in the emergency department, it is has been reported that between 6% [7] and 8.5% of patients experience a PSI [8]. In primary care, population-based record review studies have found 2–3 PSIs per 100 consultations/record reviews, with around 4% of the PSIs associated with severe harm [9].

A previous systematic review of measuring and monitoring safety in prehospital care found that a range of measures have been used (e.g. record review, incident reporting systems, surveys, and interviews/focus groups) [10]. However, the focus of this previous review was on the identification of the methods used to measure and monitor safety, and it did not establish the prevalence and harm associated with PSIs in prehospital care. A synthesis of the literature reporting the prevalence and harm associated with PSIs in prehospital will support an understanding of how often they occur and the harm they cause to patients [9]. This knowledge will support the design of safety surveillance systems that support an understanding of what is being done well in prehospital care, where improvements should be made, and support the evaluation of the effectiveness of safety interventions [10].

In order to synthesize the knowledge about prevalence and harm associated with PSIs in prehospital care, we followed the methodology used by Panesar *et al.* [9]. for assessing the prevalence and harm associated with PSIs in primary care. The aims of our systematic review were to identify how the prevalence and level of harm associated with PSIs in prehospital care is assessed; the frequency of PSIs in prehospital care; and the harm associated with PSIs in prehospital care.

## Methods

This review was planned, conducted, and reported in accordance with the Preferred Reporting Items for Systematic Reviews and Meta-Analyses guidelines [11]. The protocol was registered on PROSPERO (registration number CRD42020188401).

### Search strategy

Systematic searches were conducted across five electronic databases: Medline, Web of Science, PsycInfo, CINAHL, and Academic Search Complete. Searches were conducted in June 2020 and updated in October 2020 (see Online Supplementary Material 1 for sample Medline search strategy). Language was restricted to English, with no limits placed on the publication year. A Research Librarian assisted with the development of the search terms and protocol.

Grey literature searches were completed in October 2020. Searches were conducted across Google Scholar (first 100 returns; location set to UK), Google (first 100 returns; location set to UK), OpenGrey and Ethos.

The reference lists of included articles were manually screened to identify any additional articles potentially suitable for inclusion. Also, the reference lists of two recent reviews related to patient safety in prehospital care settings were screened [12, 13].

### Study selection

The records returned from each database were screened by one researcher (R.O.C. or K.L.), who reviewed the title and abstract of each article. The full texts of potentially eligible studies were reviewed by the research team, who made a decision about eligibility by consensus.

#### Inclusion Criteria

Included studies must report original research published between January 2001 and October 2020 and include data from prehospital care. The studies must report empirical data on one or more of the following: number/frequency of PSIs; harm associated with PSIs; include PSIs that occurred during routine care; present data in numerical form in-text; provide a usable denominator that allows the calculation of the frequency of PSIs per 100 records/patients/transports/medication doses; and be written in English. Where intervention studies indicated frequency of PSIs at multiple time points, only baseline data were extracted.

#### Exclusion Criteria

Studies were excluded if: they were not concerned with prehospital care; prehospital specific data could not be extracted; the focus was on intra- or inter-hospital patient transportation; they were concerned with non-patients, healthcare workers or others harmed in PSIs or the focus was on patients with specific medical conditions (e.g. ST-elevation myocardial infarction) or undergoing a specific procedure. Papers were also excluded if they focused on the performance of a single drug/device or a small number of specific drugs/devices; incidents that occurred under particular crisis circumstances (e.g. earthquake); incidents that were not due to the care provided in prehospital care; or incidents resulting from a healthcare provider’s decision to not perform a treatment (e.g. refraining from advanced airway management). Finally, studies were excluded if they reported qualitative or other data on PSIs in prehospital care that was non-quantifiable, or data at least partially collected in years before the cut-off date (before 2001).

### Data extraction

Data were extracted on the: country in which the study was conducted; sample size; study duration; source of data; type of prehospital care service; number of PSIs per category; the prevalence of PSIs; unit of analysis and the level of harm resulting from the incident(s). Data extraction was conducted independently by two authors (R.O.C. and K.L.). Disagreements were resolved through discussion until consensus was reached. If mutual agreement could not be achieved, a third reviewer was consulted.

### Quality assessment

Two reviewers (R.O.C. and K.L.) critically appraised each of the included studies using the Quality Assessment Tool for Studies with Diverse Designs (QATSDD [14]).

The QATSDD is a validated assessment instrument that standardizes the quality appraisal of studies with heterogeneous study methodologies. The QATSDD has previously been used in other systematic reviews related to patient safety [15–18]. The scale consists of 16 items scored on a 4-point Likert scale. All 16 items are completed for mixed-methods studies, while 14 items are completed for studies that are quantitative or qualitative in design. Scores were then summed and expressed as a percentage of the maximum possible score to allow for comparison across differing research studies [14]. Quality scores were classified according to criteria used in previous reviews utilizing the QATSDD to express that evidence was low (<50%), medium (50–80%) or high (>80%) in quality [19, 20].

### Data synthesis

As with similar reviews, descriptive and narrative synthesis of the data was conducted to identify the range in estimates and present broad trends evident in PSIs [9]. The data were too heterogeneous for pooling effect sizes. The frequency of PSIs was expressed as the number of PSIs per 100 patients/transports/medication doses and was presented in relation to ‘any type of PSI’ (i.e. a composite frequency rate of all types of PSIs) and specific PSIs (e.g. medication errors). Due to the variation in unit of measurements used across studies, medians and interquartile ranges were calculated per unit of analysis. Reviewers either extracted the frequency rate directly from the study’s text or calculated it using the data provided in the study.

Harm was coded as: no harm (any PSIs with the potential to cause harm to the patient that was either prevented or no harm was identified); low harm (incidents that required minimal additional treatment and lead to minimal or observation); moderate harm (incidents resulting in moderately increased treatment and significant harm); or severe harm (incidents resulting in permanent harm, including long-lasting physical or mental outcomes, disability or death) [21].

## Results

**Figure 1:**
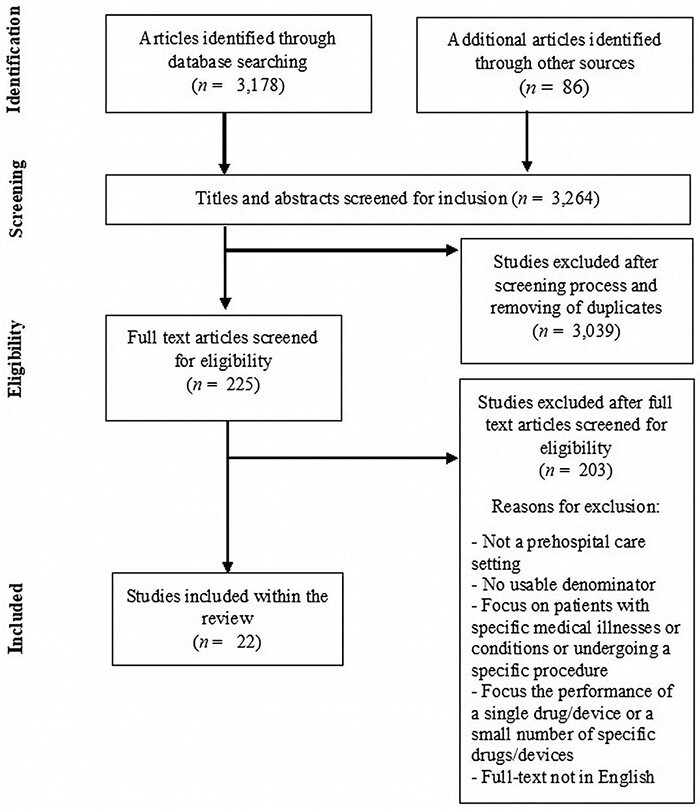
PRISMA flow diagram.

A total of 3178 articles were retrieved from the database searches, with 86 additional studies identified for full-text screening via grey literature and reference list searching (see Figure 1). In total, 225 papers were included for full-text screening, of which 22 studies [22–43] were deemed eligible for inclusion in the review. These included studies reported a total of 23 estimates of frequency of PSIs (see Online Supplementary Material 2 for more details).

### Methodological quality

Table 1 provides a summary of the quality scores. The mean QATSDD score was 25.6 (SD = 4.1, range = 16–34). Only 1 study was qualitative and only 1 study was mixed-methods in design, while the remaining 20 were quantitative. Studies generally performed well on items relating to the description of the study’s aims and objectives, the provision of detailed recruitment data and the fit between the research question and method of data collection. Studies performed poorly on items relating to the theoretical framework, consideration of the sample size and involvement of the user in the design of the study.

**Table 1:** Characteristics of included studies (*n* = 22)

Characteristics	No. of studies (%)
Study location	
Europe	12 (54.5)
North America	6 (27.3)
Asia	2 (9.1)
Australia	2 (9.1)
Prehospital service type	
Ground	15 (68.2)
Ground and air	6 (27.3)
Unclear	1 (4.5)
Source of data	
Record review (including reviews of charts, protocols, databases)	16 (72.7)
Incident reporting systems	6 (27.3)
Sample	
All patients transported	15 (68.2)
Paediatric patients only (<18 years old)	5 (22.7)
Mixed sample	2 (9.1)
Type of PSI	
Any type of PSI	10 (45.5)
Medication/prescribing incidents	8 (36.4)
Diagnostic errors	1 (4.5)
Adverse stretcher events	1 (4.5)
Suboptimal care	1 (4.5)
Deaths following prehospital safety incidents	1 (4.5)
Quality assessment	
High quality	1 (4.5)
Medium quality	19 (86.4)
Low quality	2 (9.1)

### Characteristics of included studies

The characteristics of included studies are presented in Table 1. All included studies were published between 2001 and 2020. The studies used data from incident reporting systems or a record review (see Online Supplementary Material 2 for more information). All the incident reporting systems were for use by staff, with four anonymous reporting systems [37, 41–43], and in two systems, the person submitting the report was identifiable [25, 36].

### Frequency and types of PSIs

**Figure 2:**
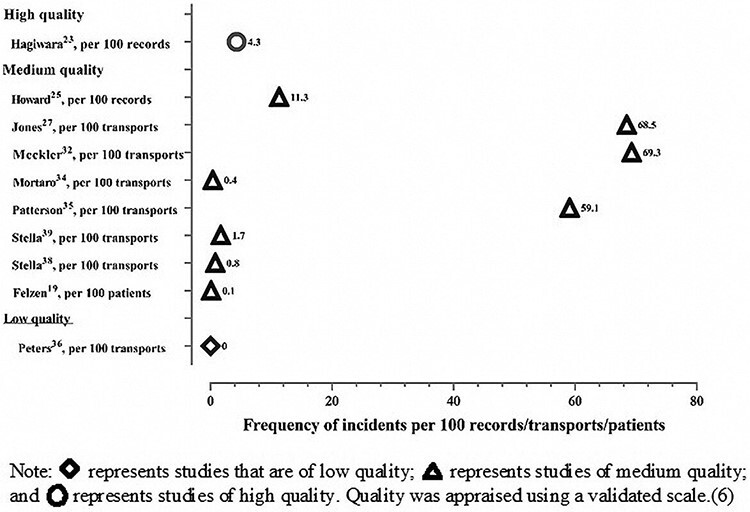
Graph of frequency of patient safety incidents in prehospital care.

Overall, 22 studies provided 23 estimates of the frequency of PSIs in prehospital care (see Online Supplementary Material 2). As presented in Figure 2, these studies reported between 0 and 71.2 PSIs per 100 records/transports/patients/medication doses, with a median prevalence of 5.9 (interquartile range (IQR), 0.6–22).

Included studies covered a variety of PSI types; most commonly measured were ‘any type of patient safety incident’ (*n* = 10; 45.5%), followed by prescribing and medication errors (*n* = 8; 36.4%), while only one study looked at diagnostic errors (*n* = 1; 4.5%), adverse stretcher events (*n* = 1; 4.5%), suboptimal care (*n* = 1; 4.5%) and deaths following prehospital safety incidents (*n* = 1; 4.5%; see Supplementary Table S2, Supplementary 2, for more information on the nature of PSIs in prehospital care).

Of the six studies that used incident reporting system data to assess the frequency of PSIs, the median number of PSIs was 0.3 per 100 transports/medication doses (IQR, 0.07–0.7). Median estimates were higher in those studies that utilized record review, which reported a median frequency of 9.9 PSIs (IQR, 4.3–34.7) per 100 records/transports/patients/medication doses.

### Harm associated with PSIs

**Figure 3:**
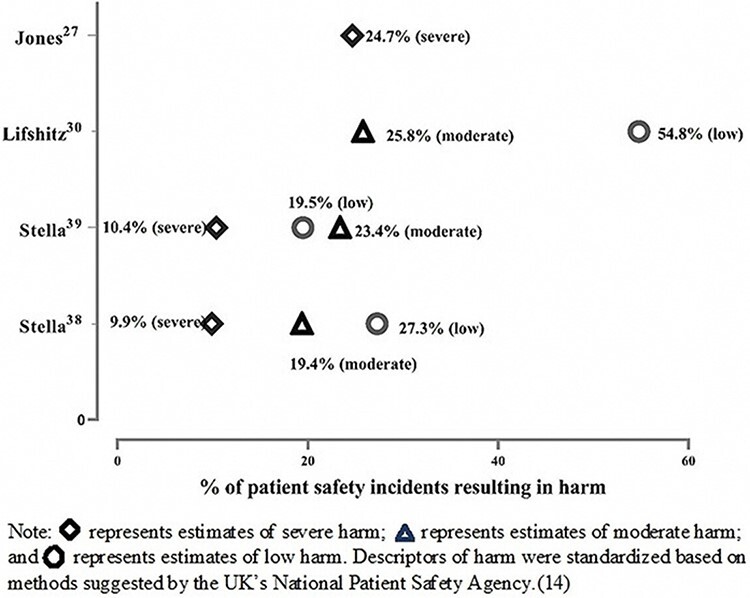
Graph of severity of harm associated with patient safety incidents in prehospital care.

Only 10 out of the 22 studies assessed the harm associated with PSIs in prehospital care. Two studies provided estimates of the potential for harm only and so were not included in the calculation of medians and interquartile ranges. The presence of harm ranged from 0% to 80.6% of PSIs, with a median of 15.6% of PSIs resulting in harm (IQR, 4.6–59%; see Supplementary Material 2 for more information). Four studies presented data on the different levels of harm (see Figure 3).

The three studies that analysed data from incident reporting systems in which harm was reported found that a median of 54.6% of incidents were associated with some level of harm (IQR, 27.3–63.3%). Studies utilizing record review data reported a median reported harm in 6.5% of PSIs (IQR, 5.6–24.7%). Only one included study assessed the preventability of PSIs and found that 45.3% of PSIs were preventable [35].

## Discussion

### Statement of principal findings

An important step in improving safety in prehospital care is a synthesis of the published literature on the prevalence of PSIs, and how often these PSIs are associated with harm. The frequency of PSIs in prehospital care was found to be a median of 5.9 per 100 records/transports/patients. A higher prevalence of PSIs was identified within studies that used record review data (9.9 per 100 records/transports/patients) as compared to incident reports (0.3 per records/transports/patients). Across the studies that reported harm, a median of 15.6% of PSIs were found to result in harm. Studies that utilized record review data reported that a median of 6.5% of the PSIs resulted in harm. For data from incident reporting systems, a median of 54.6% of incidents were associated with harm.

### Strengths and limitations

This review is a synthesis of the research available on PSIs in prehospital care. Our search strategy was comprehensive, search term development was supported by a research librarian and the search encompassed both published studies and the grey literature. The review also used the same approach as Panesar *et al.*’s [9] systematic review of safety in primary care.

The primary weakness of this review is that there is no standard for classifying PSIs in prehospital care. This means there is likely variation between studies in the definition of PSIs and the level of harm. This issue is further confounded by the impact of different data collection and classification methodologies on the prevalence and associated harm from PSIs. Our review only included data collected using record review and incident reporting systems. Although there are other methods of collecting information on PSIs (e.g. interviews [44] and surveys [45]), studies using these approaches were not represented in our review as they did not include the numerical data with a denominator required to calculate the prevalence. As only one included study [35] assessed the preventability of PSIs, it was not possible to make an assessment of the preventability of PSIs across the included studies. Finally, as with all systematic reviews, there may be a publication bias, with some types of studies more likely to be published. However, we addressed this issue through the inclusion of the grey literature.

### Interpretation within the context of the wider literature

Although incident reporting systems are widely used in healthcare, their utility for measuring safety performance is limited [46, 47]. It is well known that these systems underestimate the prevalence of PSIs [48] and overestimate the severity of harm [9]. Therefore, record reviews offer a valid method of obtaining data on PSIs—particularly when a trigger tool methodology is utilized [16]. A trigger tool methodology uses a two-stage process in which the record is initially assessed to establish whether one or more of a set of pre-established triggers are present (e.g. change in the systolic blood pressure >20% from the first measurement [28]). If one or more of the triggers are present, the record is then scrutinized in more detail to evaluate whether or not a PSI has occurred. However, despite the widespread use of a trigger tool methodology in secondary care [28], only two studies in our review used a trigger tool approach [26, 28]. It is suggested that there should be greater use of trigger tool approaches to record reviews in prehospital care.

International reviews of patient records estimate that between 4% and 17% of hospital admissions are associated with a PSI [5]. In primary care, it is estimated that there are 2 to 3 PSIs per 100 consultations/record reviews [9]. The prevalence of PSIs in the record reviews from our review is near the midpoint of the range for hospital care—despite the fact that hospitalized patients experience many more clinical encounters as compared to a patient in prehospital care. Establishing an estimate of the overall prevalence of PSIs in prehospital care is useful for highlighting the issue of patient safety and justifying resource allocation and additional research into safety [49]. However, such a broad metric of past harm fails to detect important components of safety performance and fails to provide a complete and comprehensive understanding of an organization’s safety. Therefore, there is a need to ensure that any safety surveillance system also addresses other domains of measuring and monitoring safety [50].

Interpreting the harm resulting from PSIs was challenging due to the considerable variation in how the studies described and classified harm. A similar problem regarding the classifying of harm was found in primary care PSI studies [9]. However, in addition to classifying the level of harm cause by PSIs, it is important to also obtain data on what proportion of the harm is preventable—only one of the studies in our review [35] identified preventable harm. Although there is not consensus, patient harm is classified as preventable if the cause is identifiable, modifiable, and reoccurrence can be avoided by reasonable adaption to a process or adherence to guidelines [51]. A meta-analysis of 70 studies carried out in secondary care found that 1 in 20 patients is exposed to preventable harm [52]. Therefore, as has been recommended for primary care [9], there is a need for a standardized taxonomy for classifying PSIs in prehospital care, which allows for both the classification of harm and whether or not it was preventable. This will support understanding of safety conditions and PSIs in prehospital care and assist with the development of patient safety interventions.

### Implications for policy, practice and research

The frequency of PSIs reported in this systematic review demonstrates the need for greater consideration of patient safety in prehospital care. These data emphasize the need for prehospital care organizations, and researchers, to think critically about how safety is being measured and monitored in prehospital care settings. There is a need for valid and reliable safety data in order to identify where safety could be improved and to support improvement initiatives. Record reviews may offer a valid method of obtaining prevalence data on PSIs in prehospital care—particularly when a trigger tool methodology is utilized [16, 53]. Although trigger tool methodologies are widely used to identify PSIs, there is a need for further research and assessment of the application of these methodologies to assess their reliability and validity when applied in prehospital care. Also, if these methodologies are to be used beyond research, it is important that they are easy to use with minimal training. Therefore, the usability of these tools is another important consideration for future research.

## Conclusions

The data from the record review in this systematic review has identified that 1 in 10 patients experiences a PSI in prehospital care and provides valuable insights into the prevalence of PSIs and the associated harm in prehospital care. It justifies the need to focus on safety in prehospital care to the same extent as in secondary care. It also gives direction as to how to advance methods for identifying PSIs in prehospital care and assessing the extent to which patients are harmed. This is a crucial element in advancing safety in prehospital care and supports effort to improve the safety of patients in this domain of healthcare.

## Supplementary Material

mzab138_Supp

## Data Availability

All data are either presented in the article or included in the Supplemental Material.
