# Supplementary material for: How safe is prehospital care? A systematic review
Source: Int J Qual Health Care. 2021 Oct 8;33(4):mzab138. doi: 10.1093/intqhc/mzab138 (PMC8547145; doi:10.1093/intqhc/mzab138)
Supplement: mzab138_Supp [file mzab138_supp.zip › Supplementary material 1 - search strategy.docx]

**Supplemental Material 1**

**Search strategy for Medline (OVID)**

*Search terms for prehospital care setting*

1. pre?hospital.ti,ab.
2. exp Ambulances/
3. exp Air Ambulances/
4. ambulance*1.ti,ab.
5. (emergency adj1 service*1).ti,ab.
6. (emergency adj1 transport*).ti,ab.
7. (patient* adj1 transport*).ti,ab.
8. (emergency adj1 (vehicle*1 OR car*1)).ti,ab.
9. (emergency adj1 Mobile adj1 Unit*).ti,ab.
10. (emergency adj1 dispatch*).ti,ab.
11. exp Emergency Medical Technicians/
12. (emergency adj1 technician*1).ti,ab.
13. (emergency adj1 practitioner*).ti,ab.
14. paramedic*1.ti,ab.
15. 1 OR 2 OR 3 OR 4 OR 5 OR 6 OR 7 OR 8 OR 9 OR 10 OR 11 OR 12 OR 13 OR 14

*Search terms for patient safety*

1. exp Patient Safety/
2. (patient*1 adj1 safe*2).ti,ab.
3. (safe*2 adj1 care).ti,ab.
4. exp Patient Harm/
5. (patient* adj1 harm*).ti,ab.
6. (preventable or avoidable or unnecessary) adj1 harm.ti,ab.
7. exp Iatrogenic Disease/
8. (iatrogenic adj disease).ti,ab.
9. (adverse adj1 event*1).ti,ab.
10. (sentinel adj1 event*1).ti,ab.
11. (safe* adj1 inciden*).ti,ab.
12. exp Medical Errors/
13. (health?care adj error*1).ti,ab.
14. (medica* adj error*1).ti,ab.
15. (human* adj1 error*1).ti,ab.
16. (diagnos* adj1 error*1).ti,ab.
17. (near-miss*2).ti,ab.
18. (near-failure*1).ti,ab.
19. (close-call*).ti,ab.
20. (incident* or voluntary) adj1 report*.ti,ab.
21. 16 OR 17 OR 18 OR 19 OR 20 OR 21 OR 22 OR 23 OR 24 OR 25 OR 26 OR 27 OR 28 OR 29 OR 30 OR 31 OR 32 OR 33 OR 34 OR 35

*Search terms for frequency rate*

1. exp Prevalence/
2. prevalence*.ti,ab.
3. exp Incidence/
4. inciden*.ti,ab.
5. epidemiolog*.ti,ab.
6. number*.ti,ab.
7. rate*.ti,ab.
8. frequenc*.ti,ab.
9. audit*.ti,ab.
10. 37 OR 38 OR 39 OR 40 OR 41 OR 42 OR 43 OR 44 OR 45
11. 15 AND 36 AND 46
12. Limit 47 to English language
