# Supplementary material for: How safe is prehospital care? A systematic review
Source: Int J Qual Health Care. 2021 Oct 8;33(4):mzab138. doi: 10.1093/intqhc/mzab138 (PMC8547145; doi:10.1093/intqhc/mzab138)
Supplement: mzab138_Supp [file mzab138_supp.zip › Supplementary material 2 - summary of studies about frequency UPDATED.docx]

**Supplemental material 2: Summary of studies about frequency of patient safety incidents in prehospital care**

| **Study** | **Study location** | **Prehospital service type** | **Sample** | **Type of incidents** | **Data source** | **Number analysed** | **Prevalence of PSIs** | **Severity of harm** | **Study quality** |
| --- | --- | --- | --- | --- | --- | --- | --- | --- | --- |
| Hagiwaram et al (2019) | Sweden | Ground and air | All patients | Any safety incidents | Record review | 1,080 | 4.3 per 100 records | 93.5% potential for harm (no harm)  6.5% unspecified harm | High; 81% |
| Felzen, et al (2019) | Germany | Ground | All patients | Any safety incidents | Record review | 6,265 | 0.1 per 100 patients | - | Medium; 60% |
| Howard, et al (2017) | Qatar | Ground | All patients | Any safety incidents | Record review | 159 | 11.32 per 100 records | 94.4% no harm identified  5.6% unspecified harm | Medium; 76% |
| Jones, et al (2018) | USA | Ground | Critical paediatric transports | Any safety incidents | Record review | 384 | 68.5 per 100 transports | 16.9% severe harm | Medium; 57% |
| Meckler, et al (2018) | USA | Ground | Critical paediatric transports | Any safety incidents | Record review | 378 | 69.3 per 100 transports | 76.7% potential for low harm  23.3% potential for severe harm  45.3% preventable harm | Medium; 71% |
| Mortaro, et al (2015) | Italy | Ground and air | All patients | Any safety incidents | Incident reporting system | 46,584 | 0.4 per 100 dispatches | 52% potential for low harm  28% potential for moderate harm  20% potential for severe harm | Medium; 56% |
| Patterson, et al (2012) | USA | Ground | All patients | Any safety incidents | Record review | 250 | 59.1 per 100 records | 20.3% potential for severe harm  1.4% unspecified harm | Medium; 64% |
| Stella, et al (2008) | Australia | Ground and air | All trauma patients | Any safety incidents | Incident reporting system | 4,429 | 1.7 per 100 ambulance responses | 45.5% no harm  19.5% low harm  23.4 % moderate harm  7.8% severe harm  2.6% death (severe harm)  1.3% unspecified harm | Medium; 62% |
| Stella, et al (2010) | Australia | Ground and air | All trauma patients | Any safety incidents | Incident reporting system | 56,883 | 0.8 per 100 ambulance responses | 28% no harm  27.3% low harm  19.4% moderate harm  7.3% severe harm  2.6% death (severe harm)  15.4% unspecified harm | Medium; 67% |
| Peters, et al (2014) | The Netherlands | Ground and air | Paediatric patients (<16 years) | Any safety incidents | Record review | 79 | 0 per 100 transports | - | Low; 38% |
| Galinski, et al (2018) | France | Ground | All trauma patients (>10 years) | Prescribing/medication errors | Record review | 210 | 8.6 per 100 patients | - | Medium; 55% |
| Kaufmann, et al (2018) | Germany | Unknown | Paediatric patients (<18 years) | Prescribing/medication errors | Record review | 59 | 71.2 per 100 doses | - | Medium; 55% |
| Lifshitz, et al (2012) | Israel | Ground | Adult patients (>18 years old | Prescribing/medication errors | Record review | 188 | 12.8 per 100 patients  7.1 per 100 doses | 54.8% low harm  25.8% moderate harm  19.4% no harm | Medium; 67% |
| Misasi, et al (2019) | USA | Ground | All patients | Prescribing/medication errors | Incident reporting system | 34,531 | 0.2 per 100 doses | - | Medium; 55% |
| Ramadano, et al (2019) | Germany | Ground | All patients transported | Prescribing/medication errors | Record review | 708 | 31.1 per 100 patients | - | Medium; 71% |
| Galinski, et al (2010) | France | Ground | All patients, (>16 years) | Prescribing/medication errors | Record review | 472 | 5.9 per 100 patients | - | Medium; 60% |
| Hoyle, et al (2012) | USA | Ground | Paediatric patients (<18 years) | Prescribing/medication errors | Record review | 230 | 34.7 per 100 doses | - | Medium; 62% |
| Lenssen, et al (2017) | Germany | Ground | All patients | Prescribing/medication errors | Record review | 354 | 3.2 per 100 patients | - | Medium; 55% |
| Heur, et al (2012) | Germany | Ground and air | All patients | Diagnostic errors | Record review | 596 | 9.9 per 100 patients | - | Low; 45% |
| Goodloe, et al (2012) | USA | Ground | All patients | Adverse stretcher events | Incident reporting system | 129,110 | 0.002 per 100 transports | 100% no harm (no patient injuries) | Medium; 52% |
| Mason, et al (2008) | England | Ground | Older patients (>60 years) | Suboptimal care | Record review | 1,469 | 1.1 per 100 patients | - | Medium; 64% |
| Yardley, et al (2016) | England | Ground | All adult patients | Deaths following prehospital safety incidents | Incident reporting system | 6,870,000 | 0.001 per 100 journeys | - | Medium; 62% |
